# Applications of Nanosatellites in Constellation: Overview and Feasibility Study for a Space Mission Based on Internet of Space Things Applications Used for AIS and Fire Detection

**DOI:** 10.3390/s23136232

**Published:** 2023-07-07

**Authors:** Kamel Djamel Eddine Kerrouche, Lina Wang, Abderrahmane Seddjar, Vahid Rastinasab, Souad Oukil, Yassine Mohammed Ghaffour, Larbi Nouar

**Affiliations:** 1 School of Automation Science and Electrical Engineering, Beijing University of Aeronautics and Astronautics (Beihang University), Xueyuan Road No. 37, Beijing 100191, China; 2 Algerian Space Agency (ASAL), Satellite Development Center (CDS), Ibn Rochd USTO, Bir El Djir, Oran P.O. Box 4065, Algeria; 3 Department of Electronic and Information, Beijing Institute of Technology, 5 Zhongguancun St., Beijing 100811, China

**Keywords:** nanosatellites, constellation, internet of space things, AIS, fire detection

## Abstract

In some geographically challenging areas (such as deserts, seas, and forests) where direct connectivity to a terrestrial network is difficult, space communication is the only option. In these remote locations, Internet of Space Things (IoST) applications can also be used successfully. In this paper, the proposed payload for IoST applications demonstrates how an Automatic Identification System (AIS) and a fire detection system can be used effectively. A space mission based on efficient and low-cost communication can use a constellation of nanosatellites to better meet this need. These two applications, which use a constellation of nanosatellites, can provide relevant university-level data in several countries as an effective policy for the transfer of space technology in an educational initiative project. To enhance educational participation and interest in space technology, this paper shares the lessons learned from the project feasibility study based on an in-depth design of a nanosatellite with several analyses (data budget, link budget, power budget, and lifetime estimation). Lastly, this paper highlights by experiments the development and application of a cost-effective sensor node for fire detection and the use of GPS to enable AIS capabilities in the IoST framework.

## 1. Introduction

Wildfires are more likely to occur more frequently, spread farther, and burn with more intensity when conditions are hotter and dryer. While wildfires are common in certain forest ecosystems, their frequency and severity during fire seasons are increasingly widespread. The density of wildfire activity is the greatest between 30° S and 20° N, with considerable wildfire activity occurring on more than 30% of the world’s land surface. Wildfires have a distinct diurnal cycle, even if seasonal patterns in their spread have been seen to varying degrees: The afternoon (from 1–4 pm) is typically when fire activity increases because the weather is ideal for burning (high temperatures and low humidity). Depending on the kind of combustion, the temperature of wildfires ranges from 500 to 1200 K. Many smaller, independent fire components, each with a distinct temperature, can make up a single wildfire [1]. Heavy fuel loads in forests cause fires to burn more intensely and for a longer period after the firefront has disappeared [2].

During sudden disasters, satellite imaging is crucial. Both the locations of the fires and the extent of the damage they caused are revealed. There are currently satellites that can detect fires, including traditional satellites, such as Terra and Aqua and the Landsat series, as well as smaller satellites that are presently operational or being developed [3].

The benefits of Global Navigation Satellite Systems (GNSS) are rising in sectors such as mapping, surveying, disaster warning, emergency response, transportation (air, sea, and land), and telecommunications networks [4,5]. Furthermore, satellite telecommunications have the potential as a source of information that can contribute to sustainable development for rural and remote areas in developing countries. They may help these countries to “leapfrog” stages in development by providing people access to information and helping to participate in decision-making, or more generally by improving education and health services and promoting favorable conditions for environmental protection.

Satellite communications can also be used for efficient transport management, especially maritime vessels equipped with an Automatic Identification System (AIS). AIS is an effective tool for achieving navigational safety areas, and by performing thusly, can offer serious pre-emptive maritime safety benefits, but also affords a data opportunity to understand and help to mitigate the impacts of maritime traffic on the marine environment. The existing AISs land-based receivers provide real-time data but are limited by their coverage and dependence on the network of base stations on land and vessels. Conversely, AIS satellite-based receivers can offer effective near-global coverage, but data are frequently time-delayed [6]. Satellite AIS coverage has quickly increased but is still challenged by a moderately small constellation of satellites, a limited number of ground stations to receive data, their capability to pick up a comparatively weak signal designed for earth surface use, and data integrity assuming a satellite’s footprint and overlapping transmissions [7].

Since 2008, Low Earth Orbit (LEO) satellites have been added to the combination of reception platforms and increasingly provide global data. This can comprise new types of picosatellites (0.1–1 kg), nanosatellites (1–10 kg), and micro-satellites (10–100 kg). Nanosatellites can successfully be used to track AIS radio signals in maritime areas. Additionally, the use of an AIS framework based on satellites to transmit data to assist the Internet of Things (IoT) at sea or to control ship traffic has been proposed and tested [8]. The same communication system can also be used for the IoT for inland wildfire detection and other useful applications that could be applied to develop an effective disaster safety plan. Encouraging developments in nanosatellites and advanced training on technical communications capability could allow many developing and emerging countries to place an adequate number of nanosatellites in the constellation in space for low-cost communications. To achieve real-time communication, a nanosatellite constellation is necessary to meet the requirements of this application. A good compromise is a constellation of low-cost CubeSats standards flying in different orbital planes; each CubeSat acquires a ground footprint of a chosen area in different positions and times [9]. Such constellations may not support video relay or in bulk, but can certainly support the AIS messages and instant pertinent data obtained by other IoT applications, contributing limited “telecommunication independence”.

This paper reviews the feasibility of CubeSats in the field of low-cost communications used for IoST applications, such as AIS and fire detection. The proposed CubeSat project has the following objectives:

⮚ Monitoring the marine navigation system;
⮚ Fire detection for an effective emergency plan;
⮚ Roadmap creation for the design and build of other nanosatellites able to join these proposed constellation missions;
⮚ Creation of a platform for cooperation with developing and emerging countries.

Another objective of this studied project, as the educational purpose, is to provide a hands-on experience of real nanosatellite missions to students, young professionals, research centers, and institutes dedicated to space technology.

The educational methodology and strategy are the main core of the technology transfer that will consist on:

⮚ Suggesting educational uses for real-time satellite data;
⮚ Providing data that can be interpreted by researchers and students to enrich their educational programs;
⮚ Increasing the interest and motivation of students in the development of space technology area;
⮚ Sharing field experiments between universities in different countries.

A developed technique is proposed to lead the project so that it can be implemented in educational institutions in different countries (developing and emerging countries). It is made clear that the intended educational goal, instructional technique, and experimental procedures are crucial to technology transfer because technology cannot benefit humans on its own if it is not effectively conveyed. The figure below provides a summary of this goal.

**Figure 1:**
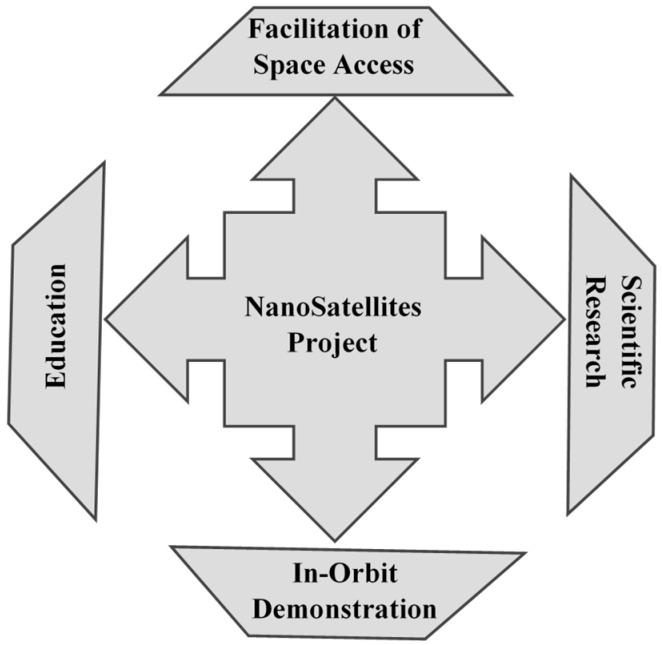
Diagram of the technology transfer.

As depicted in Figure 1, the four educational objectives for technology transfer are:

**In-Orbit Demonstration:** The objective of this project is to conduct in-orbit demonstrations of new nanosatellite technologies.

**Facilitation of Access Space:** The objective of this project is to facilitate sustained and affordable access to space by publishing new design methods and improving existing ones.

**Education and Scientific Research:** The educational objective is to enable university teams to develop and design nanosatellites, providing students with hands-on experience in satellite manufacturing and space engineering [10].

The remaining part of this paper is organized as follows: Section 2 is dedicated to the overview of IoST applications based on AIS and fire detection missions. In Section 3, an overview based on statistics about the orbits used by nanosatellites is presented. Then, the mission parameters, mission scenarios, and ground station details are also defined. The payload and subsystems of the nanosatellite with the suggested electrical architecture are discussed in depth in Section 4. The data budget, link budget, power budget, and mass budget calculations are presented in Section 5. The lifetime estimation is presented in Section 6. Lastly, Section 7 discusses the conclusion and outcomes of the planned work presented in the paper.

## 2. Overview of IoST Applications Used for AIS and Fire Detection

Satellites have emerged as an appealing alternative approach, delivering omnipresent coverage as well as critical information. Examples of their efficiency include GPS for precise locating, Earth observation satellites for monitoring environmental changes, communication satellites for worldwide connection, and their roles in emergency management and scientific research. These examples demonstrate the wide range of applications where satellite networks provide dependable coverage and critical information, cementing their place as a useful option [11]. Since the capacity and extensiveness of terrestrial networks are constrained, in isolated and disaster-affected areas, satellites are also becoming an alternative solution offering omnipresent coverage with crucial information. Furthermore, the capabilities of terrestrial IoST networks can be strategically enhanced via satellite networks. Satellites facilitate firmware updates for IoST sensor networks by broadcasting the updates over a wide coverage area. This enables remote and inaccessible IoST devices to receive timely updates, improving performance, security, and introducing new features. Using satellites reduces costs, offers scalability, real-time updates, and flexibility for diverse IoST ecosystems, enhancing the capabilities of terrestrial IoST networks [12]. The authors of [13] demonstrated the effectiveness of deploying several LEO satellite constellations for IoST applications and scenarios.

Locally and internationally, several countries want the ability to monitor ship activity using satellite technologies. International voyaging ships with a Gross Tonnage (GT), of 300 tons or more, as well as all passenger ships regardless of size, are required to be equipped with an Automatic Identification System (AIS) as of December 2004 per the International Maritime Organization’s (IMO) International Convention for the Safety of Life at Sea (SOLAS) [14]. Ships over the horizon are typically not covered by shore-based AIS receiving stations (about 50 km range). Receiving AIS signals from satellites allows for ship monitoring data to be provided as a service, offering benefits to various stakeholders in the maritime industry. Maritime authorities can improve situational awareness, enforce regulations, and enhance search and rescue operations. Cargo ships gain enhanced safety, optimized operations, and better route planning. Navies benefit from increased domain awareness, intelligence gathering, and improved coordination of task forces. Overall, satellite-based AIS services provide valuable insights and operational advantages in the maritime domain [15]. AIS is offering an opportunity to identify and be identified by others (radar affords detection, but not identity and intentions); for this reason, it was designed as a mandatory collision avoidance system for sea-going vessels. Chapter V of the SOLAS Convention mandated that vessels over 300 gross tonnages be equipped with AIS by July 2007. This requirement has been widely implemented globally, enhancing maritime safety by improving collision avoidance, search and rescue operations, vessel traffic management, and enforcement efforts. While AIS has proven effective in enhancing situational awareness and vessel tracking, its overall effectiveness may be influenced by factors such as signal range, system errors, and non-compliant vessels. Current statistics and information on the global implementation and effectiveness can be found through organizations such as the IMO and national maritime administrations [6].

**Figure 2:**
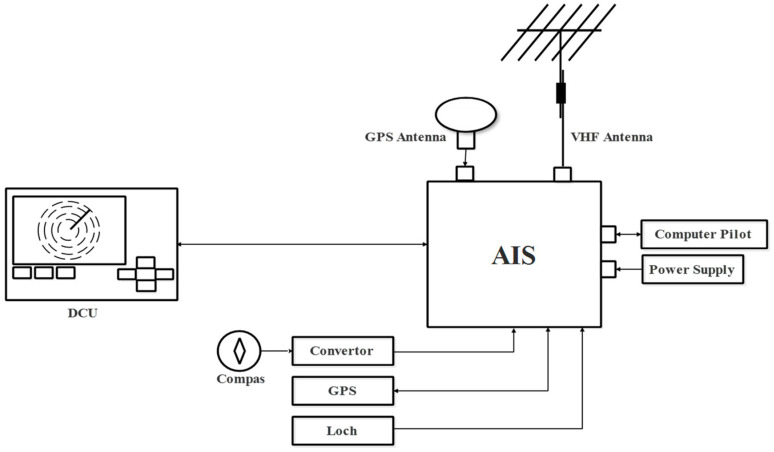
AIS architecture.

AIS is essentially an automated radio broadcast comprising data such as ship ID, location, speed, and direction of travel. The AIS, as shown in Figure 2, is composed of a transmitter, two receivers (TDMA VHF), a computer, a Digital Selective Calling (DSC) system, a satellite positioning system (GPS, GLONASS, and BEIDU), and a Display Control Unit (DCU) (control screen), and is interfaced with the vessel’s instruments (gyroscopic or satellite compass, heading change speed indicator (optional), and log).

AIS uses the two VHF frequency ranges from 161,975 MHz to 162,025 MHz, which have been reserved worldwide for this application. AIS employs the Gaussian Minimum Shift Keying (GMSK) modulation type with the rate of 9600 bauds. GMSK is a type of digital modulation that effectively encodes data by altering the carrier frequency in a way that reduces interference and spectrum spreading. It shapes the modulated signal using a Gaussian-shaped filter, which improves bandwidth efficiency and lessens fading and noise sensitivity. This modulation method enables AIS to broadcast data over a given frequency range while preserving precise and dependable connection between ships and shore stations. The packets containing 168 or 440 bits are preceded by a 24-bit preamble, making it possible to synchronize the receiver [16]. To structure and transmit data between ships and shore stations in the AIS, High-Level Data Link Control (HDLC) frames are utilized. These frames offer flow control, error detection, and synchronization for dependable communication. In order to maximize data transmission efficiency and signal integrity, AIS also uses Non-Return to Zero Inverted (NRZI) encoding, in which transitions or their lack indicate binary data. The accurate and effective interchange of information throughout the AIS network is made possible by HDLC frames and NRZI encoding working together. These HDLC-based frames also include a Cyclic Redundancy Check (CRC) code. AIS sound footprint is close to background noise, and the frames are only 30 ms long. The idea of satellite reception of AIS signals is at present being studied by a variety of projects financed by the European Space Agency and the European Commission as well as private initiative projects. The European Space Agency’s SAT-AIS initiative, for example, sought to develop and demonstrate the capacity of receiving AIS signals from spaceborne receivers [17]. NASA’s Traffic Awareness for General Aviation-Data Information Service (TAMDAR-DIS) project also included AIS reception to improve general aviation situational awareness [18]. Furthermore, collaborative activities in Canada, such as the Space-based AIS Experiment, which involved the Canadian Space Agency and industry partners, focused on showing the feasibility and usefulness of receiving AIS signals from space [19]. The RUBIN 7 and 8-AIS missions from LuxSpace/OHB, the COMDEV NTS nanosatellite mission, and the US military satellite TacSat-2 have all been conducted testing the capability of AIS from space [20]. These programs, together with research and development accomplishments by academic institutions and government agencies, jointly contribute to the advancement of AIS signal reception technology and vessel monitoring capabilities around the world. The operational nanosatellites-based AIS missions have been used as presented in Table 1.

Generally, grasslands and agricultural areas are prone to faster and less intense burning. However, it is crucial to acknowledge that wildfire behavior is influenced by multiple factors, including fuel types, weather patterns, and topography. Fuel types determine the availability and behavior of combustible materials, while environmental variables, such as wind speed, temperature, and humidity, impact fire behavior and spread. Additionally, topographical features, such as slopes and valleys, can alter the behavior and distribution patterns of a fire. Understanding the intricate dynamics of wildfires and their interactions with diverse topography requires considering these complex factors [29]. Users of satellite-based active fire detection data would desire observations to be conducted as frequently as possible, preferably hourly, with a concentration of observation times. This is because wildfires are transient but devastating in impact.

Table 2 presents a brief overview of nanosatellites performing fire detection missions.

Another fairly common use of IoST is in emergencies, such as bushfires, where first responders with environmental sensors need to communicate with the emergency management control centers. Satellites can be used to supplement network accessibility in these situations [35]. This study suggested the usage of a Sensor Network (SN) connected to an autonomous ground station for nanosatellite-based IoST applications utilized for fire detection. The SN is made up of sensor nodes, each of which has sensors for measuring the environment’s temperature, relative humidity, wind speed, wind direction, and CO_2_ level (smoke and gas sensors). When a fire is present, the ground computer (microcontroller unit) compares the measured data to certain thresholds, performs the release of an alarm, and collects some pertinent data. The sensor node’s coordinates (GPS position) are then linked to these data so that the autonomous ground station can communicate them to the space segment.

**Figure 3:**
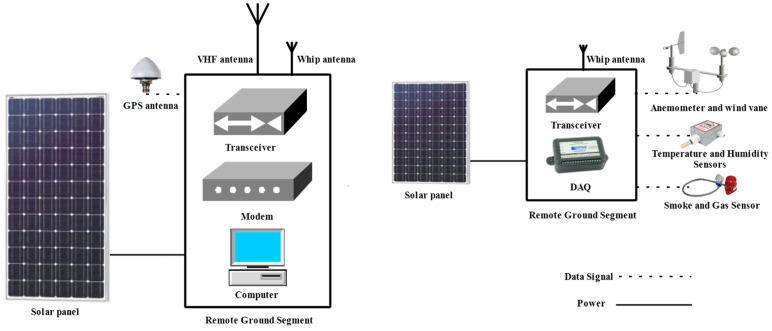
Autonomous ground segment with sensor network for fire detection.

Sending pertinent data, such as an estimate of the fire’s position, the speed, and direction of the wind, the temperature, and the CO_2_ level, is then required for agents and forest authorities to intervene and conduct the emergency plan. The effective design of the contingency plan requires the preparation of a flight plan for additional dedicated and suitable satellites (Earth observation) at the target position. Figure 3 shows the functional organization and architecture of the SN connected to the autonomous ground station.

The amount of data that can be transferred will certainly be limited by the power available at the sensor nodes, which are likely to be deployed and located in remote areas. Considering the use of low-power wireless communication protocols (WiFi, Zigbee, and LoRa) with omnidirectional antennas, the deployment of the sensor network should be optimally designed.

The computer on the autonomous ground station uses an orbitography-based software algorithm to identify the precise times the nanosatellite passes. The three elements required for this identification to be aware of and perform its calculations by this specialized software are the geographic location of this autonomous ground station, the UTC, and the NORAD–Keplerian elements. The built-in GPS receiver provides the first two components. The third component, which can be regularly updated and adjusted, is an assessment of the NORAD–Keplerian Elements of the nanosatellite.

## 3. Mission Analysis

**Figure 4:**
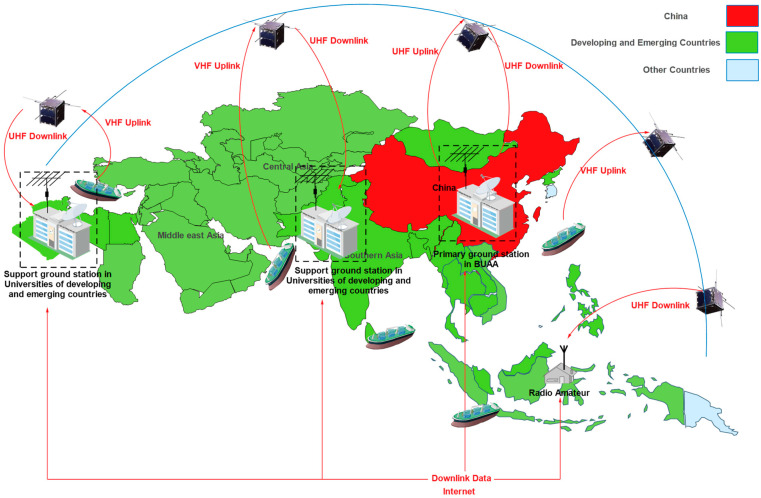
Ground/Space segment interactions for managing maritime transportation systems with the potential inclusion of other developing and emerging countries.

**Figure 5:**
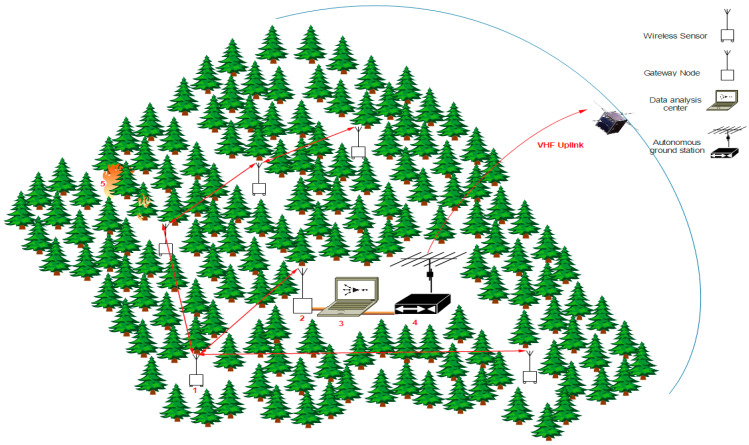
Ground/Space segment interactions for IoST application based on fire detection.

To conduct some tasks for IoST applications (AIS and fire detection) in certain regions of developing and emerging countries—South Asia, the Middle East, and North Africa—waiting times must be reduced to obtain the required temporal coverage. For the AIS mission, the straits between these countries have the densest traffic in the entire world (Malacca, Hurmuz, Bab Mandab, Suez Canal, Bosphorus, and Gibraltar). Then, for fire detection, some important forests may be targeted in Southeast Asia (in China’s mainland and Borneo rainforest), the Middle East (in Turkey, and Lebanon), and North Africa (in Algeria and Tunisia). The processes of the two proposed applications and their targeted area are illustrated in Figure 4 and Figure 5.

In these studied applications, as shown in Figure 4 and Figure 5, the nanosatellite missions are operated by the primary ground station at Beihang University. The developing countries support ground stations and the radio amateur community can receive transmitted data from the space segment. Data from support ground stations and radio amateurs can be supplied to the Beihang ground station data center via the internet. Payload users will supply payload operation requests to developing and emerging countries and will have access to their payload data stored in the Beihang data center to manage or perform the required missions.

According to [36], the revisit times for AIS decoding and fire detections are 2 h and 3 h, respectively. The revisit time for early forest fire detection should ideally be 60 min or less [37]. Table 3 presents the remaining detailed usage requirements for threshold performance of the proposed IoST missions, which will be utilized to set the operating orbit parameters.

### 3.1. Orbital Parameters LEO Design and Selection

**Figure 6:**
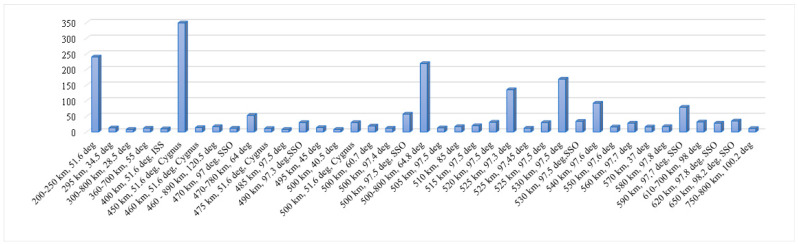
Nanosatellite number vs orbit parameters [38].

The choice of orbit parameters depends on the application used in the nanosatellite in LEO. The second criterion of the orbit is the launching opportunity because most of the nanosatellite performs a technological demonstration. An additional criterion is the constellation design that should be considered for the choice. The number of nanosatellites according to their approximate orbits after launch is presented in Figure 6.

According to Figure 6, it is obvious that a significant number of nanosatellites are launched into a 450 km circular orbit. A circular orbit with a 400–500 km interval is considered in the study presented in this paper.

The main missions’ requirements are the minimum revisit time, response time for 100% of coverage, sufficient access duration to uplink data, and low cost for every mission. The most appropriate orbit for the proposed missions is the LEO, which can be around 400 km of altitude. Based on these requirements, the continuous monitoring of AIS and fire detection over some regions of developing and emerging countries should be guaranteed as the main objectives of the proposed missions. For example, the two main areas of focus are the coverage provided by the AIS mission in the South China Sea, which can be helpful for many emerging and developing countries (maritime silk route), and the fire detection mission scheduled to cover forest areas in mainland China.

Several designs of nanosatellite constellations dedicated to Earth coverage are conceivable; nevertheless, they are unlike in coverage duration and revisit time. Each of the cases of studies was obtained by calculations from the developed model based on the literature [39] and verified by using Analytical Graphics Inc.’s Satellite Toolkit (STK), Inc. The analysis focused on three parameters: revisit time, access duration, and response time for 100% of coverage.

The constraints that must be taken into consideration while choosing the orbital elements for a successful mission analysis include the type of space mission, the various types of the constellation, and the potential for the launch to cross various orbits. Deploying nanosatellites to suitable orbits can be challenging due to their restricted launch capabilities, which force them to be transported into orbit as an afterthought aboard bigger spacecraft. An extensive orbital study based on the actual launch opportunity is necessary to decide whether a launch is appropriate for the constellation. Therefore, the circular orbit of 400 km of altitude is taken into consideration based on the available launch opportunity after numerous iterations using the trial and error method to select the ideal orbit parameters. Then, it is necessary to incline the orbit to 49 degrees to approach the southeast of the Chinese mainland, located between 110–129° east longitude and 29–48° north latitude, where forests are present [40], and the South China Sea, located between 107–114° east longitude and 4–17° north latitude, where ship navigation is extremely congested. This covers the majority of the forest areas and navigation areas in eastern and southern China. The orbital elements of the LEO circular orbit chosen for this study are listed in Table 4.

**Figure 7:**
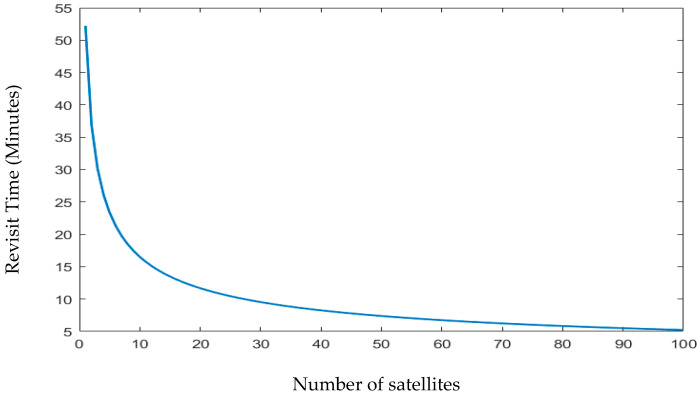
Revisit time versus the number of satellites.

According to [41], for covering a specific area of low-latitude ground, a higher number of nanosatellites in a circular LEO orbit provides better coverage with good performances. Figure 7 shows the plot of the revisit time as a function of the number of satellites in the constellation.

**Figure 8:**
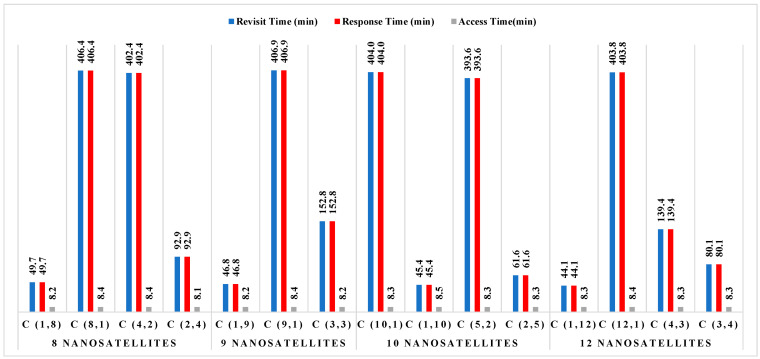
Performance results of each constellation for areas in the South China Sea.

Using the findings from Figure 7, it is common practice to use between 8 and 12 satellites to produce a revisit time for a nanosatellite constellation of between 15 and 20 min. There are several possible designs for nanosatellite constellations that are intended to cover the earth and maritime zones; however, they differ in terms of revisit time and response time for 100% of coverage. Figure 8 illustrates the performance outcomes (revisit time, response time, and access time) for various constellation configurations for South China Sea regions. The constellation distribution characteristics are depicted in this figure as C (number of nanosatellites in each orbit and number of orbit plans).

Figure 8 illustrates that, for all constellation’s configurations with various numbers of nanosatellites, the average access time for constellations in the South China Sea is around 8 min. According to the threshold performance requirements provided in Table 3, these data are acceptable and are probably the best technique to estimate transmission duty cycles; however, no final judgment has been taken on that matter to date. Defining access periods further and selecting a duty cycle and transmission time that maximizes the opportunity to obtain data are possible future tasks. The data shown in this figure indicate that the constellation of 12 nanosatellites distributed over 12 orbital plans performs exceptionally well in terms of revisit time and response time (according to Table 3). For this constellation, the maximum revisit duration and response time is 44.1 min. Nevertheless, the performances attained with a constellation based on eight orbital plans are still appropriate with only eight nanosatellites. For any clear-cut viewpoint, a compromise between several nanosatellites’ costs and performances—specifically, their tolerable revisit and response times—is the best option.

**Figure 9:**
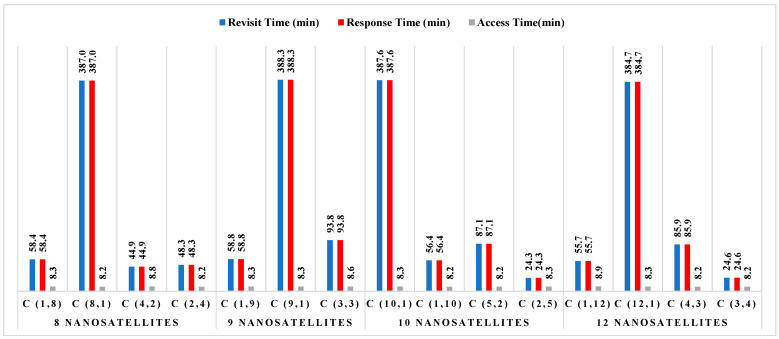
Performance results of each constellation for the forest in the Chinese mainland.

Figure 9 displays the findings of each constellation’s performances for the forest on the Chinese mainland, similar to Figure 8, where C (number of nanosatellites in each orbit and number of orbit plans) represents the constellation distribution parameters.

A constellation of 12 nanosatellites placed in the 12 orbital planes can be the most appropriate solution to be used to achieve 100% coverage, as shown in Figure 9, while considering the threshold performance requirements listed in Table 3. For this constellation, the maximum revisit duration and response time is 55.7 min. It might be possible to also revisit time according to the threshold performance requirements shown in Table 3 by forming a constellation of eight nanosatellites in eight orbital planes. To arrive at the appropriate mission analysis decision, as a compromise method, depending on the required and shorter revisit time (on the order of at least less than one hour), combinations of a reduced number of nanosatellites with constellation configuration were considered.

### 3.2. Mission Scenarios

**Figure 10:**
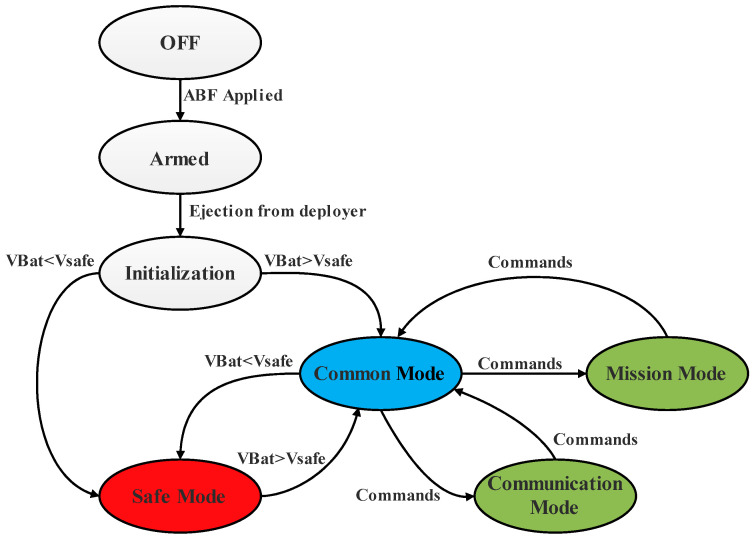
Proposed missions’ modes for the nanosatellite.

The diagram overview of the five modes was selected as the operation modes for these proposed missions of nanosatellites and is presented in Figure 10.

**Initialization mode:** once the nanosatellite is ejected from the deployer, the status of the nanosatellite is checked in the early stage of nanosatellite operation; the antenna is deployed; the communication and power systems are initialized.

**Common mode:** The nanosatellite orbits with no mission or communication. Therefore, in this mode, only the OBC and EPS are activated.

**Safe mode:** once the power supply is insufficient, the nanosatellite is operated with minimum functions and minimum power, where only the EPS is activated.

**Mission mode:** The nanosatellite performs its missions, such as receiving data of the specified areas using a mounted payload based on AIS. In this mode, the nanosatellite accomplishes its missions by exploiting its payload with the full activation of OBC and the function of ADCS.

**Communication mode:** The data obtained from the mission are communicated to the ground station. Telemetries data are also sent to the ground station and remote commands are received from the ground station.

### 3.3. Ground Station

The ground station is scheduled to be situated at Beihang University (39.9824° N, 116.3488° E) for the nanosatellite visibility analysis. On the one hand, backup ground stations can be employed to maintain radio contact if the primary ground station (Beihang University) faces a problem and will be unable to function. Commands may also be allowed from these redundant ground stations for the continuity of the operations plan. On the other hand, additional amateur radio stations may be permitted to access, download, and decode data from some nanosatellites. As a result, the frequencies and operational modes of this nanosatellite may be made public for this purpose.

**Figure 11:**
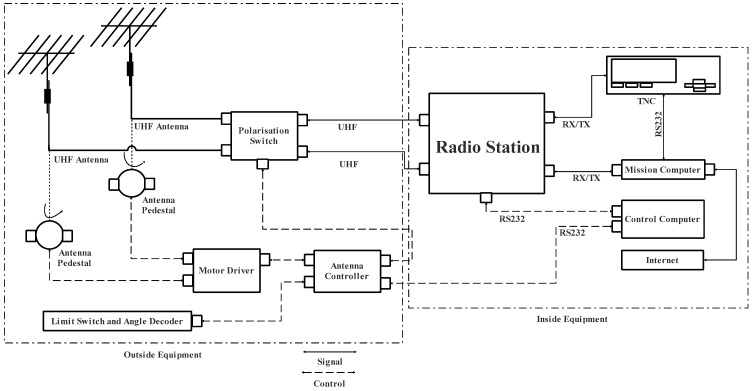
Ground station block diagram.

**Figure 12:**
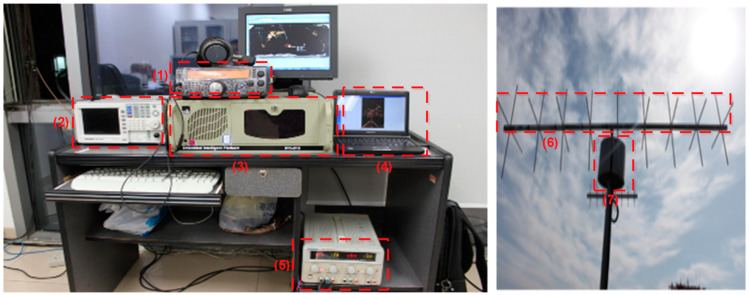
BUAA Beihang Ground Station Implementation: (1) radio station, (2) TNC, (3) polarization switch, (4) mission computer, (5) power supply, (6) VHF/UHF antenna, and (7) antenna pedestal.

The designed ground station is composed of a UHF/UHF ground station and can be located in the satellite center of Beihang University (BUAA). Figure 11 shows this ground station block diagram and Figure 12 shows its actual implementation with the Yagi antenna.

As shown in Figure 12, the ground station at BUAA is a modular system that is installed specifically for nanosatellites employing UHF radio frequencies in LEO. Using a steerable antenna system, the ground station can autonomously track certain nanosatellites. All hardware (polarization switch, moto driver, antenna controller, radio station, and TNC), as well as specialized software, were included in the complete installation in the mission computer and control computer.

## 4. Payload and Subsystem Architectures and Design

**Figure 13:**
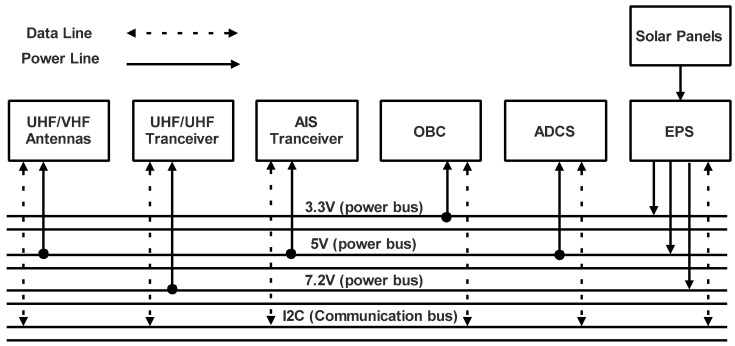
Electrical architecture of the proposed nanosatellite platform.

In this section, the working function of the proposed payload and subsystems of the nanosatellites’ platform are presented and explained. The main payload for the proposed missions (AIS and fire detection) consists of a communication transceiver. This proposed payload consists of a VHF receiver (161.975 and 162.025 MHz, respectively) to collect data on AIS and fire detection. The remaining components of the platform are made up of different subsystems. Another independent UHF transceiver-based communication subsystem is in charge of sending data to the ground station and receiving commands. Furthermore, an On-Board Computer (OBC) that manages data and executes commands for the mission plan is used. To guarantee a constant supply of energy to the nanosatellite, according to the electrical specification of the nanosatellite platform, an appropriate Electrical Power Subsystem (EPS) is used. Finally, an Attitude Determination and Control Subsystem (ADCS) is also proposed to stabilize the nanosatellite so that it can conduct the mission effectively. The electrical architecture of the proposed nanosatellite platform is shown in Figure 13.

Details of the payload and subsystems for the proposed nanosatellite platform are presented in the following subsections.

### 4.1. Payload Based on VHF Receiver

The receiver payload for this mission is essentially a dual-channel VHF receiver that can be tuned to any of the VHF channels in the maritime VHF band between 156.025 and 162.025 MHz and for an autonomous ground station that will use the VHF band between 144 and 146 MHz to send data gathered from a sensor network. The specifications of this type of receiver are presented in the following Table 5.

**Figure 14:**
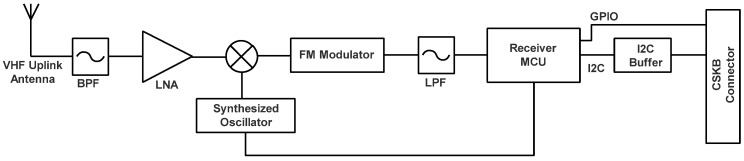
VHF uplink block diagram used as payload.

The block diagram of the proposed VHF receiver can be seen in Figure 14.

This proposed communication payload, which is intended to collect measurements from AIS transmitters and the autonomous ground station attached to the sensor network used for fire detection, can be in-house customized and manufactured or provided by space companies such as ISIS or SatLab [42,43]. For example, the SatLab can provide a space- and flight-tested commercial solution for this AIS application [44]. The CubeSat Space Protocol (CSP) is supported by the receiver provided by SatLab, which can greatly shorten the buy-to-fly time and facilitate integration. Three CSP servers, which are needed to configure the receiver, read back status, download messages, and raw samples, are present in this flight-proven receiver. The Blob Transfer Mechanism (BTP), a remote shell and compact file transfer protocol, is based on CSP. BTP supports file uploading, download, and standard file operations, including list, delete, copy, and move.

### 4.2. Platform Transceiver

The selected UHF uplink/UHF downlink transceiver is a full-duplex communication system for nanosatellite TT&C applications. This transceiver can operate in commercial bands of the UHF frequency spectrum. It is low-power, low-mass, and highly configurable, offering the flexibility of changing data rates and frequencies in flight. This space-qualified transceiver was designed and tailored for nanosatellite missions and cross-compatible with other subsystems, such as onboard computers and antenna systems. The specifications of this type of transceiver are presented in Table 6.

**Figure 15:**
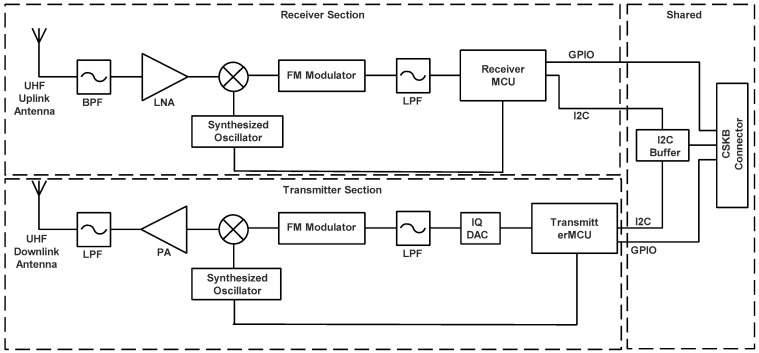
UHF uplink/UHF downlink transceiver block diagram.

From the above table, the technical configuration based on the block diagram of the selected transceiver is shown in Figure 15.

The downlink of mission data and detailed telemetry data are handled by the UHF band transmitter at a maximum of 500 kbps. The transceiver selected for the nanosatellite can be provided by the ISIS manufacturer and will be its communication system bus [45]. These functions are enabled by an omnidirectional whip antenna and dipoles antenna mounted on the bottom of the nanosatellite, such as the antenna provided by ISIS [46].

### 4.3. Electrical Power Subsystem

**Figure 16:**
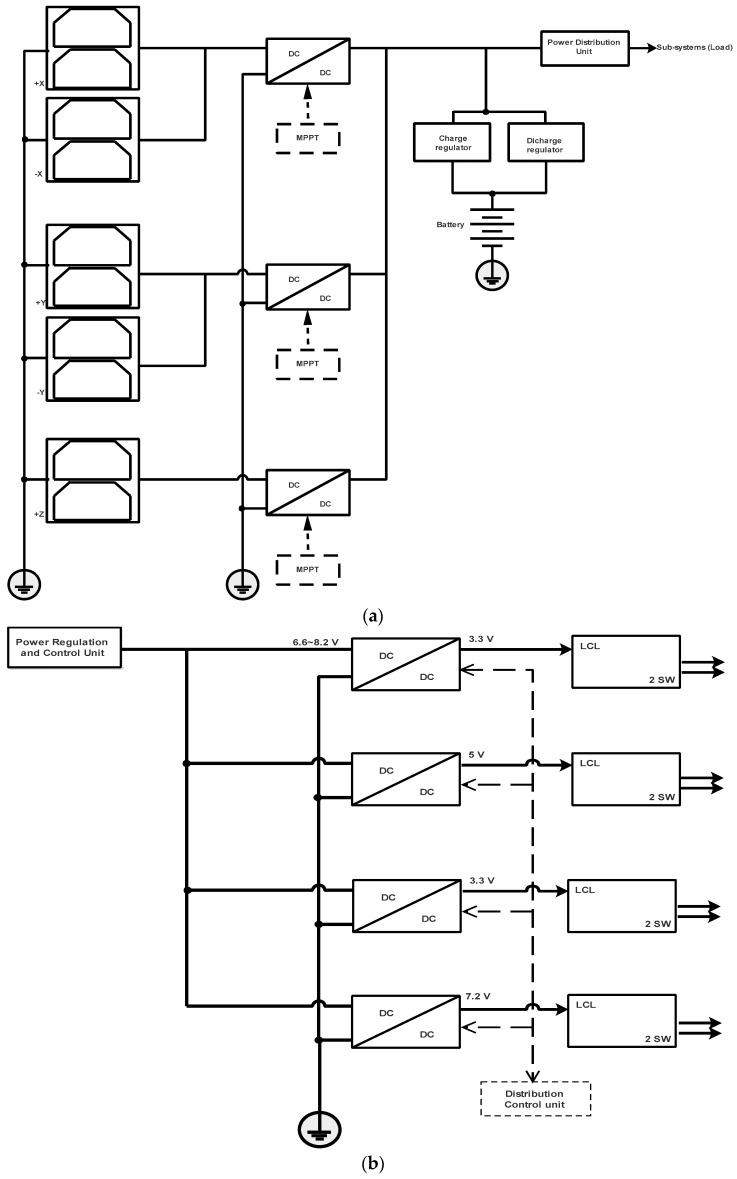
Proposed EPS configuration for CubeSat with open solar panels structure: (**a**) power regulation and control unit, and (**b**) power distribution unit.

In the proposed nanosatellites’ EPS, shown in Figure 16a, the solar panels are body -mounted and their opposing faces are coupled to the same power converter (MPPT1 for the −X array and +X array, MPPT2 for the −Y and +Y array, and MPPT3 for the −Z and +Z array). The power converters can be connected in parallel, and each converter has a Maximum Power Point Tracker-implemented method (MPPT) and a Battery Charge Regulator (BCR). The module of the power distribution and protection circuits based on LCL is presented in Figure 16b.

Using the above topology also functions as a built-in redundancy: the loss of a single boost converter or damage to a solar panel will not deactivate the whole EPS. There is no requirement to regulate the main bus, as the subsystems themselves will separately regulate their specific supply [47]. A similar topology is used in commercial and developed CubeSat EPS, as presented in [48,49,50,51,52,53].

### 4.4. On Board Computer

OBC uses a high-performance and robust design, 400 MHz, power-efficient ARM9 processor-based MCU. This compatible OBC with standard CubeSat components is available from ISIS [54]. The computer was developed for nanosatellite data handling and ADCS processing. In this OBC, the FreeRTOS operating system for simple and lightweight cooperative multitasking is used.

The chosen OBC features the following performances as standard:

⮚ Volatile Memory: 64 MB SDRAM;
⮚ Code Storage: 1 MB NOR Flash;
⮚ Critical Data Storage: 512 kb FRAM;
⮚ Mass Data Storage: 2 × 2 GB high-reliability SD cards for fail-safe data storage (up to 32 GB on request) or 2× any size standard SD cards;
⮚ A total of 2× redundant Real Time Clock (RTC);
⮚ I2C, SPI, and UART interfaces to another OBC;
⮚ On-board temperature sensor;
⮚ External onboard watchdog and power controller.

**Figure 17:**
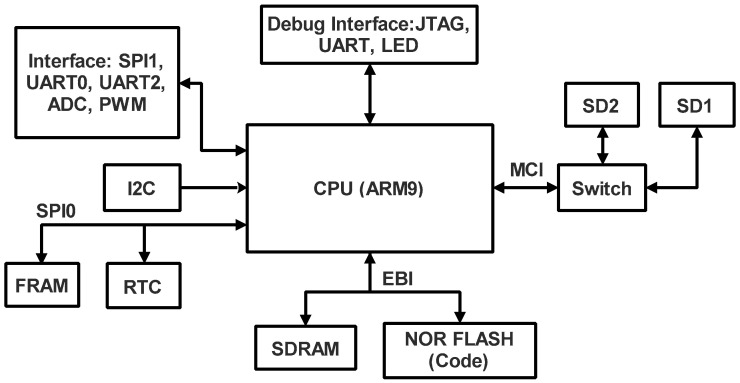
OBC block diagram.

The schematic of this OBC configuration is shown in Figure 17.

The controlling unit of some ADCS tasks can be embedded in this low-power OBC, where it runs estimator and control algorithms, logs TLM, and manages communication to the ADCS modules.

### 4.5. Attitude Determination and Control Subsystem

**Figure 18:**
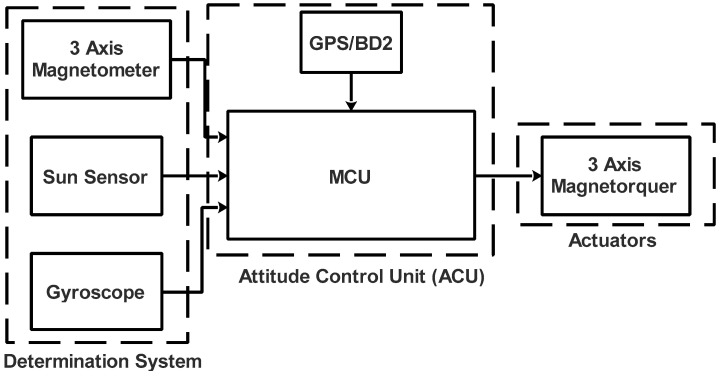
ADCS block diagram.

The architecture of the ADCS proposed in the nanosatellite to accomplish the proposed mission is presented in the block diagram of Figure 18.

In this ADCS configuration, the MagneTorquers (MTs) used are integrated into solar panels. This type of Embedded MTs was chosen because of its low power consumption. As mentioned previously, the MCU is integrated into high-performance and low-power 32-bit ARM9.

## 5. System Engineering Analyses

This section elaborates on budget calculations for data, link, power, and mass to confirm the viability of the suggested nanosatellite platform for conducting the intended missions (AIS and fire detection).

### 5.1. Data Budget

A significant and noteworthy amount of data are produced by the VHF receiver-based payload that is utilized to receive information from the AIS and the autonomous ground station connected to the fire detection sensor network. For the AIS application, a message of 256 bits is transferred at a rate of 9600 bits per second utilizing a binary Gaussian minimum shift keying (GSMK) modulation during each of the brief 26.67 millisecond slots that the AIS uses to broadcast information. The ships can transmit their message bursts in any of the 2250 slots that make up each frame.

For fire detection application, the data budget is dominated by the measurement of Global Positioning System (GPS) coordinate (24 bits, which are enough for good accuracy) to locate where the fire is propagating with some measurements of smoke, temperature, wind speed, and direction (10 bits of resolution for each measurement). Given these data specifications and numbers, the maximum data rate requirements are close to 64 bits for one autonomous ground station.

According to the analysis and platform specifications, it is feasible to access the ground after five or six orbital rotations, and OBC sends requests for telemetry data every 5 s. These parameters should be considered when planning the estimation of the data budget as shown in Table 7. 

As mentioned in Section 5.2, the size of the OBC and transceivers technology was chosen to support data sizes and data rates up to 64 MByte and 9600 bps, respectively, which allows the mission to have a large safety margin in the data budget. This data budget estimate is adaptable to the mission strategy and open to various revisions.

### 5.2. Link Budget

A link budget is an analysis tool that is used to establish whether or not a communication link meets such mission requirements by considering data and factors such as transmitted signal power, frequency, data rate, and link bandwidth. During this calculation procedure, the gain and loss of the Radio Frequency (RF) signal from the modulation at the transmitter to the demodulation at the receiver are considered. The nanosatellite link can be affected by different factors, such as ionospheric and atmospheric attenuation, which generates influence on the polarization of the wave, losses that result from the misalignment of the antennas and losses of the free space depending on the tilt range since it is crucial for the geometric analysis of tracking. In the same way, the noise of the system that is directly related to the temperature and the noise of the different elements in a decisive way in the calculations are taken into consideration.

#### 5.2.1. Downlink Budget

Table 8 displays the calculated parameters required for conducting a downlink budget analysis.

#### 5.2.2. Uplink Budget

The calculated parameters necessary for conducting uplink budget analysis are shown in Table 9, Table 10 and Table 11.

### 5.3. Power Budget

This study recommends an EPS configuration that satisfies mission criteria and maintains nanosatellite functionality throughout the mission. It will be more than enough for the mission, if the EPS nanosatellite can adequately power the aforementioned subsystems, such as:

⮚ On-Board Computer (OBC);
⮚ Communication system (Transceiver (Tx) and Receiver (Rx));
⮚ Attitude Determination and Control System (ADCS);
⮚ Payload (AIS and VHF receiver for SN).

The power consumption of these above-defined subsystems is shown in Table 12.

Table 13 presents the power consumption of the different mission modes (see Section 4). In this table, the power magnitudes of each subsystem are categorized as minimum or maximum.

**Figure 19:**
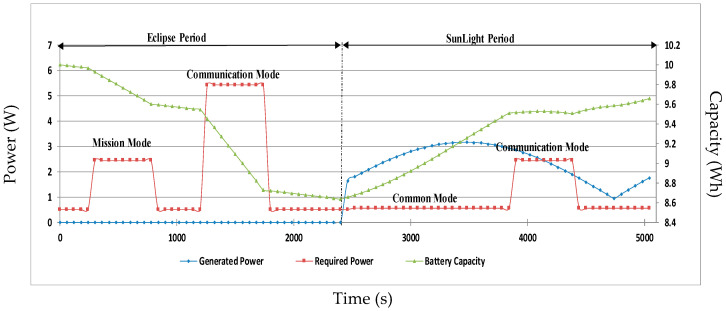
Profiles of power consumption, power generation, and battery capacity.

After many iterations, the worst-case power consumption will be considered for the rest of the analysis for mission feasibility. According to ECSS standards and SMAD, a margin from 5% to 20% or 25% has to be applied to meet the mission power budget used for sizing, depending on the level of the design maturity [39,55]. Based on the different power consumption modes of the nanosatellite, Figure 19 shows, during one orbit, the simulated baseline scenario of the power consumption with 20% of the margin, power generation, and capacity profiles. In this simulation, the mission mode and the two different communication modes (Tx and Rx) are considered, which means a worst-case orbit scenario.

To ascertain whether EPS can withstand the mission, it is necessary to consider a rough assumption made when estimating the power budget in a preliminary mission study. However, there may be many adjustments to whatever subsystem is activated, how it will operate (maximum or minimal), and for how long it will be activated, depending on the concept of operations and mission plan.

### 5.4. Mass Budget

The mass budget shown in Table 14 lists estimates for the contribution of various components to the mass of the nanosatellite.

A mass margin of approximately 120.5 g is obtained, according to Table 14. The predicted mass budget respects the total mass constraint, which was set at less than 1.33 kg.

## 6. Lifetime Estimation

**Figure 20:**
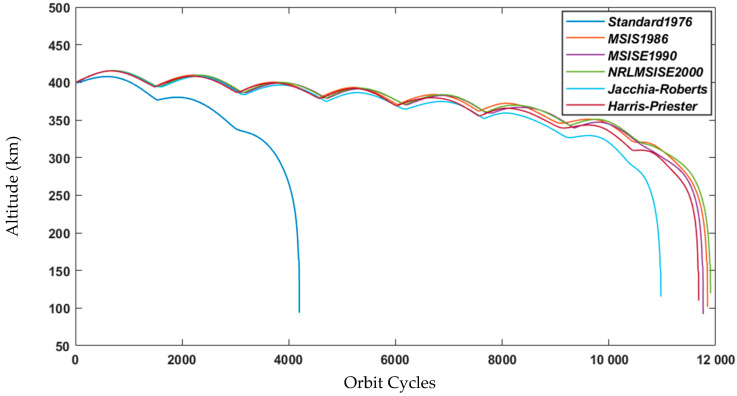
Altitude decay according to the orbit cycles.

The orbital decay’s primary influencing characteristics (orbit altitude and mass) for the proposed nanosatellite were taken into consideration as precisely as possible to eventually obtain a reliable prediction of lifetime (orbit decay), which is displayed in Figure 20.

Figure 20 compares the lifetime predictions for six different atmospheric models, including the widely used Jacchia–Roberts, the most evolved NRLMSISE 2000, and the oversimplified Standard 1976. When the findings of two atmospheric density models are compared, the disparities are obvious. As illustrated below, the standard 1976 model is systematically overly pessimistic and should be avoided. With the NRLMSISE 2000 model, the nanosatellite’s lifetime is predicted to be roughly two and a half years for the 400 km CO. The practical instance is obtained by employing the Jacchia–Roberts algorithm, which provides a lifetime of around two years.

**Figure 21:**
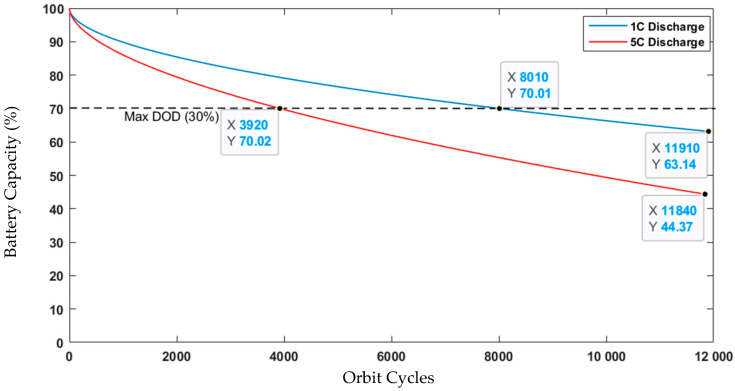
Battery capacity degradation according to the orbit cycles.

A crucial step in determining the viability of the nanosatellite mission and its lifetime is the reliable assessment of the battery lifetime to orbital cycles and discharge capacity. If the battery is discharged faster, its lifetime will be reduced, as the results of the following analysis, shown in Figure 21, demonstrate the feasibility of the proposed mission.

According to the results presented in Figure 21, it can be noticed that the battery can resist up to 1.5 years, with 1C of battery discharge, due to limitations imposed by DoD-rated value, which is 30%.

## 7. Experimental Results of the Proposed Low-Cost Sensor Node Architecture and AIS

**Figure 22:**
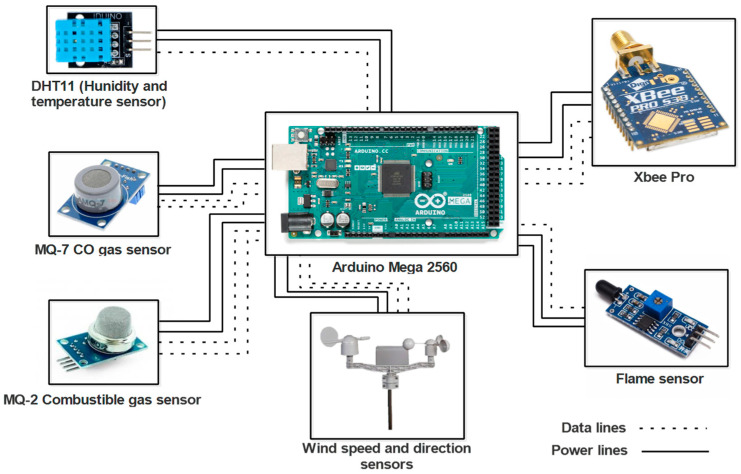
Low-cost sensor node architecture.

This section presents the experimental results of the proposed low-cost sensor node architecture and AIS for use in a nanosatellite, which is designed for IoST applications as previously discussed. The architecture, shown in Figure 22, is based on low-cost sensors and includes Arduino Atmega 2560 as the data acquisition system and Xbee pro as the communication system within the sensor network.

**Figure 23:**
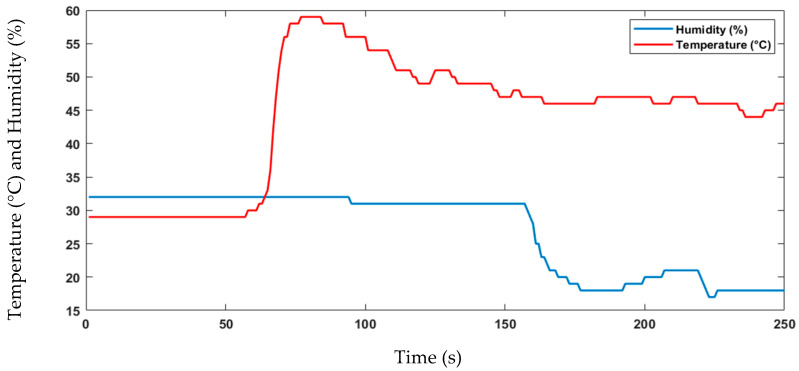
Data of the temperature and humidity changes observed in the experiment.

**Figure 24:**
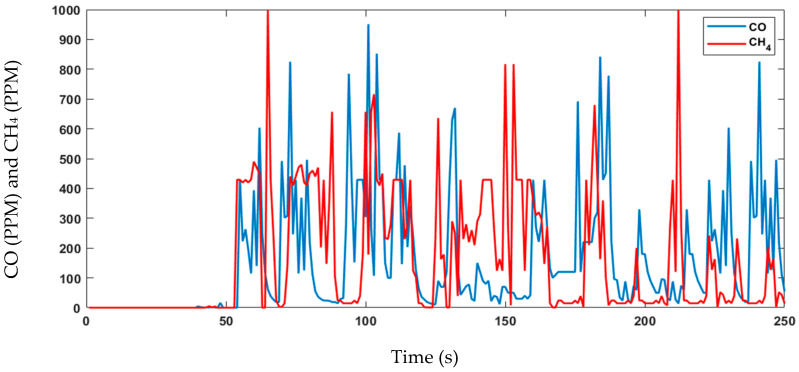
Data of the detection of gases at different levels in the experiment.

**Figure 25:**
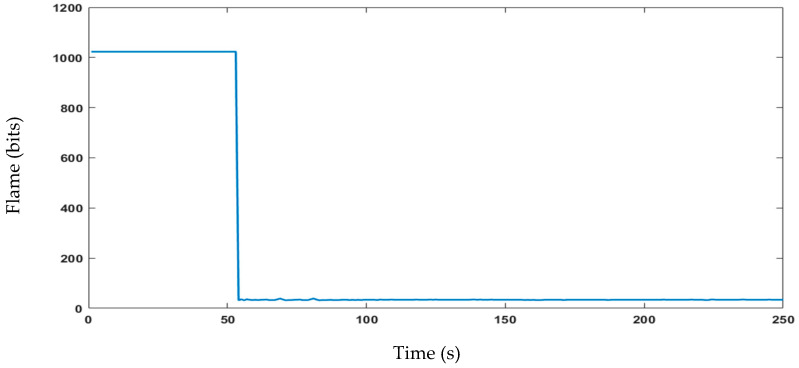
Data of flame level detection in the experiment.

**Figure 26:**
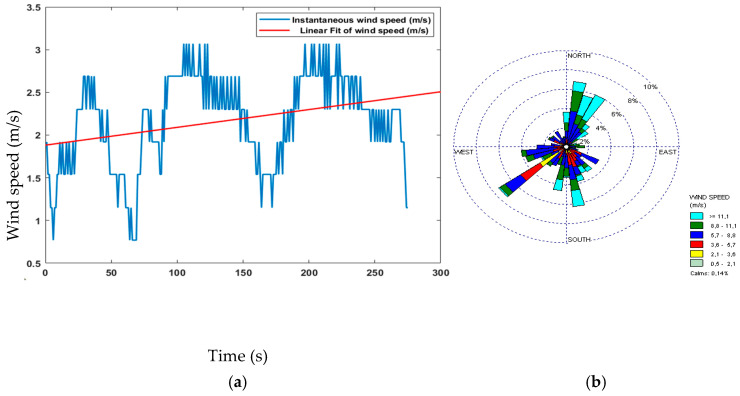
(**a**) Wind speed and (**b**) wind direction.

The proposed sensor node architecture includes the KY-026 sensor, specifically designed as a flame sensor to detect flames within a wavelength ranging from 760 nm to 1100 nm. In addition, forest fires release gases, such as CO_2_, CO, CH_4_, and VOCs, including benzene, toluene, and formaldehyde. To detect the released gases by forest fires, CO and CH_4_ are commonly monitored using the MQ-7 sensor, although it should be used in conjunction with other sensors and monitoring techniques. The MQ-2 sensor can detect various flammable gases, smoke, and VOCs, making it another useful tool for forest fire detection. Temperature and humidity sensors can also help to predict and prevent fires by detecting increases in temperature and low humidity levels, respectively. The DHT11 sensor is an example of a digital temperature and humidity sensor that can be used for forest fire detection. Combining these sensors with other detection technologies can create a more comprehensive forest fire detection system. The experimental data are shown in Figure 23, Figure 24, Figure 25 and Figure 26. During this four-minute experiment, significant variations in the experimental data were observed in these figures both before and after the occurrence of a fire at 50 s.

The fire decision can be reached after the analysis of the above results, such as temperature, humidity, gas, and flame presence as criteria. The alarm threshold is based on a simple decision procedure according to the measurement data and determines a fire. In this experiment, the recorded temperature surpassed 50 °C, indicating the presence of a fire and the flame observed was at a lower level compared to previous measurements, further confirming the occurrence of a fire. The other sensor node measurements data are important to detect fire presence and deduce the causes. The wind speed measurements do not show the presence of fires but can aid in emergency response and firefighting planning.

**Figure 27:**
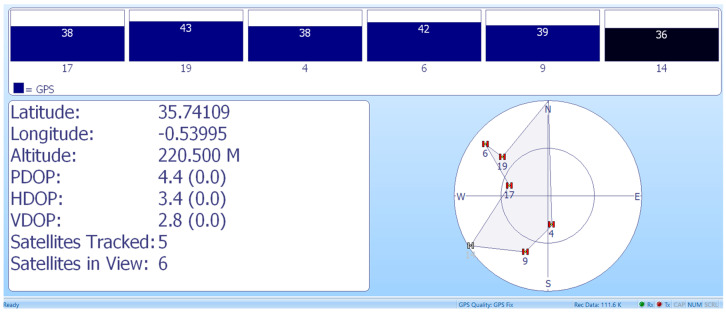
Position coordinates obtained by GPS used for AIS.

In the application of AIS, the GPS dedicated to maritime application was used to show the coordinates, as presented in Figure 27.

## 8. Conclusions

Building efficient services based on nanosatellites is a praiseworthy objective, especially in light of the inefficiency and lack of infrastructure in terrestrial communication networks. Therefore, the authors presented a project proposal, in this paper, for carrying the nanosatellite challenge and its technology transfer to developing and emerging countries. Furthermore, the communication system embedded in these nanosatellites in a constellation can have the ability to provide technical information based on the Internet of Space Things (IoST) to generate solutions for different applications, such as Automatic Identification Systems (AIS) and fire detection. The proposed technology within this space project was initially developed as a collision prevention tool by AIS, intending to track data (global vessel locations, informing the movement of vessels over time) collected by nanosatellites from maritime transportation systems. The second application has the crucial capability of monitoring wildfire processes and their consequences on ecosystems, the environment, and global warming. Therefore, the mission objective of the nanosatellite project is to develop an effective and functional constellation of Low Earth Orbit (LEO) nanosatellites specifically designed to address the aforementioned purposes. The proposed constellation aims to overcome the limitations of a single nanosatellite by leveraging multiple satellites working in concert, enabling improved revisit times and enhancing the overall efficiency and functionality of the system.

Meeting pre-established criteria, such as accomplishing the required revisit time, access duration, and response time to assure 100% of coverage of the areas of interest through the nanosatellite constellation, proved the viability of the planned mission. Additionally, the findings of the budget analysis (data, link, power, and mass) and estimations of lifetime offer convincing proof that the suggested nanosatellite platform is well-suited to successfully conduct the necessary missions (AIS tracking and fire detection).

Based on the experimental findings, it is clear that the suggested low-cost sensor node architecture and AIS technology effectively proved the feasibility of fire monitoring and marine safety. The system’s integration of numerous sensors, such as temperature, humidity, gas and fume sensors, flame, wind speed, and direction sensors, allows for the reliable identification of fire occurrences and offers important information for effective emergency preparedness. Furthermore, the successful testing of GPS technology shows AIS localization’s significant contribution to maritime safety measures and the protection of marine ecosystems. These experimental findings provide persuasive proof of the proposed technology’s enormous potential in increasing fire monitoring, disaster preparedness, and maritime navigational procedures, ultimately leading to a more secure and sustainable future within the IoST.

Furthermore, establishing a strong strategy among countries to a forge long-lasting cooperation between the academic community and the rising industries in this sector could lead to high competence in space technology projects. Consequently, this space project is open to students and engineers from different countries. In this context, problem-based learning (PBL), which has been effectively applied at Beihang University, can be thought of as the use of educational and technical methodologies. PBL can undoubtedly assist to create a network of scientists interested in space technology. Three main levels of operation are possible for this project: production and field deployment of applications, education, and research. These levels are connected to foster cooperation within the educational space program.

Future studies may include designing a larger nanosatellite platform, such as a 3 U or 6 U, for pollution monitoring (using an atmospheric spectrometer) and the study of atmospheric and environmental factors.

## Figures and Tables

**Table 1:** Operational AIS embedded on nanosatellites.

	CAT-3	ZACube-2	AAUSAT-4	AISSat 2	AISAT	AAUSAT-3	AISSat 1	CanX-6 (NTS)
Operator	Universidad Politécnica de Cataluña [21]	Cape Peninsula University [22]	Aalborg University [23]	UTIAS [24]	DLR [25]	Aalborg University [26]	UTIAS [27]	UTIAS [28]
NanosatMass (kg)	9	4	0.88	6	14	0.8	6	6.5
Size	6 U	3 U	1 U	-	1 U	1 U	-	2 U
Power Consumption	-	-	1.15 W	0.97 W	15 W	1.15 W	0.97 W	5.6 W
Launch date	-	2017	2016	2014	2014	2013	2010	2008
Payload	AIS + High resolution VIS and VNIR camera	AIS + Low resolution NIR imager	AIS	AIS	AIS	AIS	AIS	AIS

**Table 2:** Operational fire detection payload embedded on nanosatellites.

	TUBIN	Spire Nanosat	Lume-1	FIREBIRD	CSIROSat-1
**Operator or contractor**	Technische Universität Berlin [30]	OroraTech [31]	l’Université de Vigo [32]	DLR [33]	Commonwealth Scientific and Industrial Research Organisation (CSIRO) [34]
**Nanosatellite Mass (kg)**	17	9	2.1	15	4
**Size**	-	6 U	2 U	two 1.5 U CubeSats	3 U
**Power Consumption**	-	-	-	-	-
**Launch date**	2017	2021	2018	2012	Not launched, but expected in 2022
**Payload**	Infrared microbolometer	Thermal-infrared camera	Software-defined radio (SDR) and HUMPL	Imaging multi-spectral radiometers (vis/IR)	Detector of invisible infrared light

**Table 3:** Threshold performance requirements for IoST based on AIS and fire detection.

	Acceptable	Desired
**Access duration for AIS**	7.5 min	More than 8 min
**Access duration for Fire Detection**	7.5 min	More than 8 min
**Revisit time for AIS**	2 h	Less than 1 h
**Revisit time for Fire Detection**	3 h	Less than 1 h
**Response time for AIS**	2 h	Less than 1 h
**Response time for Fire Detection**	3 h	Less than 1 h

**Table 4:** Chosen initial orbital elements.

Orbit Type	CO
**Semi-major axis**	A = 6771 km
**Inclination**	I = 49°
**Eccentricity**	E = 0.0017
**RAAN**	0°
**Argument of periapsis**	0°
**True anomaly**	0°

**Table 5:** Specifications of the AIS receiver.

Parameter	Value
**Power Supply**	5 V DC
**Power Consumption**	<1 W (receiver only during full load)
**Mass**	55 g
**Dimensions**	90 × 96 × 15 mm
**First Receiver Frequency Range**	156.025–162.025 MHz
**Second Receiver Frequency Range**	144–146 MHz
**First and Second Receiver Modulation Scheme**	Gaussian Minimum Shift Keying (GMSK)
**First and Second Downlink Data Rate**	9600 bps
**Channel Bandwidth**	RSSI of 100 kHz
**Operating Temperature Range**	−30 to +70 °C

**Table 6:** Specifications of the selected UHF Uplink/UHF downlink transceiver.

Parameter	Value
**Power Supply**	6.5–20 V DC
**Power Consumption**	4 W (transmitter on), 0.48 W (receiver only)
**Mass**	75 g
**Dimensions**	90 × 96 × 15 mm
**Transmitter Frequency Range**	267–273 MHz
**Transmitter Power**	27 dBm
**Transmitter Modulation Scheme**	Binary Phase Shift Keying (BPSK) with G3RUH scrambling
**Transmitter Data Rate**	1200, 2400, 4800, and 9600 bps
**Receiver Frequency Range**	312–322 MHz
**Receiver Sensitivity**	−104 dBm Sensitivity for BER 1E-5
**Receiver Modulation Scheme**	Frequency Shift Keying (FSK) with G3RUH scrambling
**Downlink Data Rate**	9600 bps
**Protocol**	AX.25 or HDLC
**Operating Temperature Range**	−20 to +60 °C

**Table 7:** Data budget of the proposed nanosatellite mission.

AIS Data Budget	
**Maximum payload frame size**	72 Kbyte
**Data rate download of communication radio**	9600 bps
**Time to download 1 payload frame**	60 s
**Fire Detection data budget**	
**Maximum payload frame size (from 100 nodes)**	800 byte
**Data rate download of communication radio**	9600 bps
**Time tso download 1 payload frame**	666.67 ms
**Telemetry data budget**	
**EPS (10 bits of resolution for each measurement)**	120 bits
**ADCS (10 bits of resolution for each measurement)**	120 bits
**GPS**	24 bits
**Thermal (10 bits of resolution for each measurement)**	120 bits
**Orbital Period**	5063.45 s
**Communication time interval**	5 s
**Total telemetry budget**	48.6 Kbyte
**Data rate download of communication radio**	9600 bps
**Time to download 1 frame of telemetry**	40.5 s
**Total time to download AIS data frame + telemetry frame**	100.5 s
**Total time to download fire detection data frame + telemetry frame**	41.17 s

**Table 8:** Downlink budget to BUAA ground station.

Parameter	Value
**EIRP (dBW)**	−3.1
**Rcvd. Frequency (GHz)**	0.27
**Rcvd. Iso. Power (dBW)**	−149.81
**Flux Density (dBW/m^2^)**	−139.73
**g/T (dB/K)**	−7.1
C/No (dB*Hz)	72.07
**Bandwidth (kHz)**	19.2
**C/N (dB)**	29.24
**Eb/No (dB)**	9.6
**G.S. Rcvr (dB)**	20.1
**S/N required (dB)**	9.6
**BER**	1.0 × 10^−3^
**System Link Margin (dB)**	10.5

**Table 9:** Uplink budget of BUAA ground station.

Parameter	Value
**EIRP (dBW)**	34.9
**Rcvd. Frequency (GHz)**	0.32
**Rcvd. Iso. Power (dBW)**	−117.56
**Flux Density (dBW/m^2^)**	−106.09
**g/T (dB/K)**	−25.92
C/No (dB*Hz)	84.97
**Bandwidth (kHz)**	3.00
**C/N (dB)**	50.20
**Eb/No (dB)**	19
**BER**	1.0 × 10^−3^
**System link Margin (dB)**	30.4

**Table 10:** Uplink budget of autonomous ground station connected with the sensor network.

Parameter	Value
**EIRP (dBW)**	32.51
**Rcvd. Frequency (GHz)**	0.14
**Rcvd. Iso. Power (dBW)**	−117.29
**Flux Density (dBW/m^2^)**	−112.61
**g/T (dB/K)**	−25.9
C/No (dB*Hz)	85.27
**Bandwidth (kHz)**	3.00
**C/N (dB)**	50.5
**Eb/No (dB)**	45.45
**BER**	1.0 × 10^−3^

**Table 11:** Uplink budget of AIS.

Parameter	Value
**EIRP (dBW)**	21.76
**Rcvd. Frequency (GHz)**	0.16
**Rcvd. Iso. Power (dBW)**	−128.88
**Flux Density (dBW/m^2^)**	−123.23
**g/T (dB/K)**	−25.88
C/No (dB*Hz)	73.7
**Bandwidth (kHz)**	3.0
**C/N (dB)**	38.93
**Eb/No (dB)**	33.88
**BER**	1.0 × 10^−3^

**Table 12:** Power consumption of the subsystems.

Subsystems	Minimum Power	Maximum Power
**EPS**	120 mW	160 mW
**OBC**	-	400 mW
**TT and C**	480 mW	4000 mW
**ADCS**	1005 mW	1125 mW
**Payload**	-	480 mW

**Table 13:** Consumed power according to the different operation modes.

Modes	Minimum Power	Maximum Power	Duration
**Common mode**	520 mW	560 mW	68.4 min
**Mission mode**	2025 mW	2065 mW	8 min
**Communication mode**	2025 mW	4520 mW	8 min

**Table 14:** Mass budget for the proposed nanosatellite.

Components	Mass (g)
**Chassis**	155
**Solar panels**	50 × 5
**ADCS**	351
**EPS (with accumulators)**	163
**Communication System**	75
**Payload**	55
**OBC**	56
**Antenna**	85
**Harnessing**	15
**Margin**	±10%
**Total**	1325.5

## Data Availability

Not applicable.
